# Assessing the Impact of a Vi-polysaccharide Conjugate Vaccine in Preventing Typhoid Infections Among Nepalese Children: A Protocol for a Phase III, Randomized Control Trial

**DOI:** 10.1093/cid/ciy1106

**Published:** 2019-03-07

**Authors:** Katherine Theiss-Nyland, Mila Shakya, Rachel Colin-Jones, Merryn Voysey, Nicola Smith, Abhilasha Karkey, Sabina Dongol, Dikshya Pant, Yama G Farooq, Kathleen M Neuzil, Shrijana Shrestha, Buddha Basnyat, Andrew J Pollard

**Affiliations:** 1 Oxford Vaccine Group, Department of Paediatrics, University of Oxford, United Kingdom; 2 Oxford University Clinical Research Unit–Nepal, Patan Hospital, Kathmandu; 3 Center for Vaccine Development and Global Health at the University of Maryland, Baltimore, MD; 4 Paediatric Research Unit, Patan Hospital, Kathmandu, Nepal

**Keywords:** typhoid vaccine, randomized control trial, protocol, Nepal

## Abstract

**Background:**

Enteric fever is estimated to affect 11–20 million people worldwide each year. Morbidity and mortality from enteric fever primarily occur in lower-income countries, with children under 5 years of age experiencing a significant portion of the burden. Over the last few decades, the control of enteric fever has focused primarily on improved water and sanitation, with the available vaccines unsuitable for children and primarily used by travelers. A new typhoid conjugate vaccine (Vi-TCV), prequalified by the World Health Organization (WHO) and highly immunogenic in children under 5, has the potential to reduce the typhoid burden in endemic countries.

**Methods:**

This study is a double-blinded, randomized, controlled trial with a 2-year follow-up to assess the protective impact of the Vi-TCV vaccine, compared with a control vaccine, in children from 9 months to 16 years of age. The primary outcome of interest is the reduction in the number of culture-confirmed typhoid cases attributable to Vi-TCV. Approximately 20 000 children living in the Lalitpur district, within the Kathmandu valley, will be enrolled in the study and followed to measure both safety and efficacy data, which will include adverse events, hospitalizations, antibiotic use, and fever frequency.

**Results:**

Both the intervention and control vaccines are WHO prequalified vaccines, which provide a health benefit to all participants. Children have been chosen to participate because they bear a substantial burden of both typhoid morbidity and mortality in this population. The results of this study will be disseminated through a series of published articles. The findings will also be made available to the participants and the broader community, as well as local stakeholders, within Nepal.

**Conclusions:**

This is the first large-scale, individually randomized, controlled trial of Vi-TCV in children in an endemic setting, and will provide new data on Vi-TCV field efficacy. With Vi-TCV introduction being considered in high-burden countries, this study will support important policy decisions.

**Clinical Trials Registration:**

The trial is registered on the ISRCTN registry (for details, see https://doi.org/10.1186/ISRCTN43385161; registry number: ISRCTN 43385161).

Enteric fever, including typhoid fever, is a systemic illness caused by the human restricted pathogens *Salmonella enterica* serovars Typhi and Paratyphi. It is estimated to affect 11–20 million people worldwide annually, with an estimated 100 000 to 200 000 fatalities per annum, primarily in lower-income countries with poor quality water and inadequate sanitation [1, 2]. Areas with an incidence of >100/100 000 are considered endemic, including many countries in Africa, South Asia, South-East Asia, and Central Asia [3]. The burden of disease and mortality is increasingly recognized in the under 5 age group, as well as in older children and young adults [4–8]. Enteric fever also remains a concern in high-income countries, for travelers to endemic regions and laboratory workers [9, 10].

Control of typhoid fever, historically, has been achieved primarily through improved water and sanitation infrastructure, leading to the elimination of disease as a public health problem from most developed countries. While improved sanitation is the most effective solution to preventing widespread typhoid, there are substantial costs and difficulties in implementing these measures rapidly in resource-poor settings. As such, the use of an effective vaccination program targeting the highest-risk populations will likely be a useful and cost-effective addition to control measures [8]. Given the causative organisms are human-restricted, global eradication is possible and an effective vaccine can support elimination efforts.

Previous efforts to control typhoid through vaccination have had limited success [2, 11]. 3 typhoid vaccines have previously been licensed: the inactivated whole-cell vaccine, the polysaccharide vaccine (Vi-PS), and Ty21a [11]. Vi-PS and Ty21a are currently available, but have failed to be used routinely in endemic typhoid countries, due to either age restrictions and/or a lack of feasibility (multiple doses) [2, 6, 11–16]. A prior typhoid conjugate vaccine (TCV) (Vi-rEPA), was highly efficacious in a clinical trial in Vietnam and was shown to be immunogenic in infants, but was never commercialized [17, 18].

Vi-TCV (Tybar-TCV) is a newly available vaccine developed by Bharat Biotech and consisting of 25 µg of Vi polysaccharide antigen conjugated to a tetanus toxoid carrier protein. Vi-TCV induces a T-cell–dependent response. It can, therefore, induce antibody responses in infants and young children under 2 years of age and has the potential to generate durable immune responses via the induction of immunological memory.

A Phase III, randomized, controlled trial comparing Vi-TCV with Vi-PS in sub-groups receiving boosters of either vaccine at 2 years demonstrated significantly higher anti-Vi immunoglobin G (IgG) titres in the Vi-TCV group compared with the Vi-PS group in participants aged 2 years to 45 years (titres of 1685.3 EU/ml [95% confidence interval {CI} 1468–1797] in Vi-TCV vs 445.6 EU/ml [95% CI 323–615] in Vi-PS) [19]. An open-label trial conducted alongside this assessed the immunogenicity of a single dose of Vi-TCV in infants and toddlers aged 6–23 months and demonstrated seroconversion to anti-Vi IgG [19]. Safety data from the same study demonstrated that Vi-TCV was well tolerated by all age groups [20].

Efficacy data are available from a recent controlled human infection study at the University of Oxford [21]. The study measured the efficacy of single doses of Vi-TCV, Vi-PS, and a control vaccine against typhoid infection after an oral challenge. The study was conducted in healthy, United Kingdom adult volunteers and the challenge was performed 28 days after vaccination. Using a composite diagnostic endpoint of clinical and/or microbiologically-confirmed typhoid fever, the Vi-TCV and Vi-PS vaccines demonstrated comparable protective efficacies of 54.6% (95% CI 26.8–71.8%) and 52.0% (95% CI 23.2–70.0%), respectively, when compared with the control vaccine [21]. When applying a definition of typhoid fever which more closely approximates diagnoses in health-care settings—that is, fever, followed by confirmatory bacteraemia—in a post hoc analysis, the protective efficacy of the Vi-TCV vaccine was 87.1% (95% CI 44.2–96.9%), compared with 52.3% (95% CI -4.2 to 78.2%) for the Vi-PS vaccine[21].

The data from seroconversion and efficacy studies are strong, with 1 study showing a seroefficacy of 85% (95% CI 80–88%) [22]. Large-scale field impact studies for Vi-TCV, demonstrating a reduction in the burden of disease attributable to typhoid infections, have not yet been conducted, and this vaccine is currently not used routinely in Nepal.

Vi-TCV is licensed for use in India and Nepal, and has been WHO prequalified for broad use [23]. Despite this, TCV is still not available for use in Nepal through routine vaccination or at private clinics. This study will assess the impact of Vi-TCV in a field setting in order to inform and support the use of the vaccine as a control measure for typhoid fever in endemic settings, and to reduce global morbidity and mortality from typhoid fever. Vi-TCV has shown promise from existing studies; it can produce seroconversion in infants; it potentially produces long-lasting immunity; and it is efficacious in a controlled challenge setting. As such, it is an obvious candidate to test in a field impact study. The primary objective of this trial is to determine how effective Vi-TCV is at preventing blood culture–confirmed symptomatic infections caused by *S.* Typhi.

Our secondary objectives are to investigate the safety outcomes associated with Vi-TCV; determine the impact of vaccination with Vi-TCV on the incidence of admission rates for fever; measure the number of days spent in hospitals from febrile illnesses; measure the differences in rates of hospital/clinic presentations for febrile illnesses; measure paratyphoid infection rates; and measure the incidence of clinically-suspected enteric fever. Some of the exploratory objectives are to measure self-reported and/or prescribed antibiotic/antimicrobial use for inpatients/outpatients; determine the effect of vaccination on growth and weight in children <5 years of age; determine the immunogenicity of Vi-TCV and the persistence of antibodies induced by Vi-TCV; and measure all-cause mortality and all-cause mortality with fever.

## METHODS AND ANALYSIS

### Study Design

This is a participant- and observer-blind, individually randomized, controlled trial with a 2-year follow-up to assess the protective impact of Vi-TCV.

### Population

Children aged 9 months to <16 years at the time of enrollment will be eligible to participate. Participants will be identified as living within the defined catchment area of the Lalitpur district, within the Kathmandu valley, Nepal. Trial staff, including local community health volunteers, will identify households with children <16 years of age and approach them for participation.

This age range has been selected because children bear a substantial burden of the disease in both mortality and morbidity, without an effective vaccine available in the routine vaccination schedule. Therefore, this demographic group has the most to gain from vaccination with Vi-TCV and would be the primary target for any subsequent vaccination campaign.

#### Inclusion Criteria

To be included in the study, the following inclusion criteria must be met:

1. Parent/legal guardian is willing and competent to provide informed consent. If the participant is 7 years of age or older, assent will also be sought.
2. Participant is aged between 9 months and <16 years (ie, up to 15 years and 364 days) at the time of vaccination.
3. Participant is in good health on the day of vaccination.
4. Parent/legal guardian confirms that they/their child will be willing and able to comply with the study requirements, including follow-up contacts, according the schedule (Table 1).
5. Participant lives within the study catchment area at the time of vaccination.

#### Exclusion Criteria

Any of the following criteria will be grounds for exclusion:

1. Participant has knowingly received a typhoid vaccine in the last 3 years.
2. Participant has a known allergy to any of the vaccine components.
3. Participant or parent/guardian has any medical or social reason that will prevent the participant from conforming to the study requirements, as judged by a medical professional.
4. Participant or parent/guardian is planning to move away from the catchment area within the next 6 months.

### Study Setting

The study setting of the Lalitpur district, within the Kathmandu valley, Nepal, has been chosen because enteric fever is endemic to Nepal, with a high incidence in Kathmandu. The Strategic Typhoid Alliance Across Africa and Asia has conducted passive surveillance of typhoid and paratyphoid incidences in the study area of Kathmandu, providing baseline typhoid rates for comparison [24].

Typhoid has been recognized locally as a public health concern, and there is political support and interest for the introduction of a new typhoid vaccine for children.

### Intervention and Randomization

Vi-TCV will be the intervention vaccine, and a Meningococcal serogroup A vaccine (MenAfriVac) against *Neisseria meningitidis* serogroup A (MenA), manufactured by the Serum Institute of India, will be the control vaccine.

Participants will be randomized in a 1:1 ratio to receive either the Vi-TCV or MenA vaccine, using stratified block randomization with randomly varying block sizes from 6–12. Stratification will be by age (9 months to <5 years old or ≥5 years old to <16 years). Participant randomization will occur at the vaccination visit, using a purpose-built, offline, mobile application. Individual logins will be given to unblinded staff, which will allow secure access to the application. No trial staff will be able to make changes to randomization information stored on the application.

**Figure 1.**
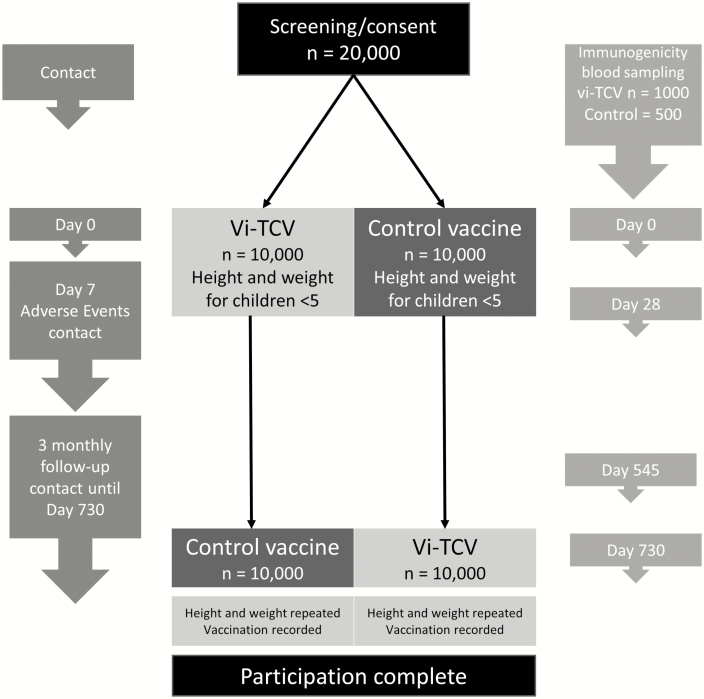
Flow chart of planned trial participant contact. Abbreviation: TCV, typhoid conjugate vaccine.

In addition, for those who consent to blood collection, the same mobile application will be used to randomly select 1500 individuals to be included in an immunogenicity sub-study. Blood sampling randomization will be on a 2:1 basis (1000 Vi-TCV and 500 MenA) and be age stratified (<5 years and ≥5 years). A diagram of planned trial activities can be seen in Figure 1.

In order to maintain blinding, randomization and vaccine preparation will take place behind a curtain, where only unblinded staff are permitted. Follow-up contacts will not be undertaken by unblinded staff (Colin-Jones & Shakya et al, *in progress in this supplement*). Unblinding will take place after the 2-year follow-up period has been completed.

Portable servers, accessible through an Intranet, will be set up in each clinic and data will be directly entered onto laptops connected to a local server [Tracy and Farooq, *in progress in this supplement*]. The local server will be transported to a facility with an Internet connection daily, and the data collected during that day will be uploaded onto the main server.

### Passive Surveillance and Participant Follow-up

**Figure 2.**
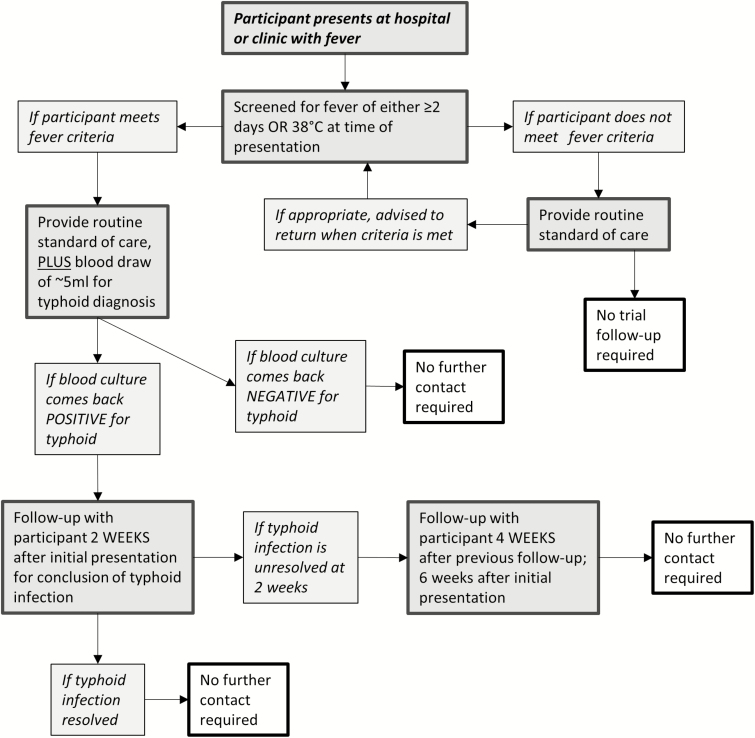
Unscheduled procedures flow chart.

Passive surveillance will be conducted at the local hospital and in community-based health clinics. Trial participants who present with ≥2 days of subjective, persistent fever or a temperature of at least 38 degrees C at presentation will be tested for typhoid via blood culture. Enteric fever cases will be treated with antibiotics and followed for resolution (Figure 2). Hospitalization, surgical procedures for perforation, and other serious outcomes will be followed and recorded. Participants will be contacted every 3 months to follow up on recent fevers, antibiotic use, and other health outcomes of interest (Table 1). Medical record reviews will be performed if a participant follow-up contact identifies a fever and/or hospitalization.

**Table 1.** Visit and Sample Schedule

Visit	1	2	3	4	5	6	7	8	9	10	11^a^
Day	0	7	28	90	180	270	365	455	545	635	730
Permissible time window (days)	…	+7/-1	±4	±14	±28	±28	±56	±56	±56	±56	±90
Screening	X	…	…	…	…	…	…	…	…	…	…
Consent	X	…	…	…	…	…	…	…	…	…	…
Randomization	X	…	…	…	…	…	…	…	…	…	…
Vaccination	X	…	…	…	…	…	…	…	…	…	…
Medical history and exam	X	…	…	…	…	…	…	…	…	…	…
Blood collection^b^	X	…	X	…	…	…	…	…	X	…	X
Height and weight^c^	X	…	…	…	…	…	…	…	…	…	X
Follow-up contact^d^	…	X^e^		X	X	X	X	X	X	X	X
Vaccination with either Vi-TCV or MenA^f^	…	…	…	…	…	…	…	…	…	…	X
Documentation of vaccine receipt^g^	X	…	…	…	…	…	…	…	…	…	X

Abbreviations: AEFI, adverse event following immunisation; MenA, *Neisseria meningitidis* serogroup A; TCV, typhoid conjugate vaccine.

^a^Ideally, all of the visit 11 activities will occur simultaneously, but the follow-up contact may occur separately, if necessary.

^b^Blood sampling for immunogenicity will be in a subset of 1000 Vi-TCV and 500 control participants. The planned blood draws are ~5 ml per visit. The total maximum volume will be ~20 ml per participant. The blood draw on Day 0 will occur before vaccination; the blood draw at Day 545 will aim to occur at the same time as the follow-up; and the blood draw at Day 730 will occur at the same time as unblinding and vaccine documentation.

^c^Height and weight will be measured only in children under 5 years of age at the time of enrollment.

^d^Follow-up contact includes: ensuring the participant and family still live in the area and are happy to continue with the study; inquiring regarding work and school absenteeism; recording mortality and morbidity in the participant, including fevers; and providing a reminder to attend a trial health-care facility if they develop a fever of ≥2 days.

^e^At 7 days, full AEFI reporting will be collected during the follow-up contact.

^f^Either Vi-TCV or MenA vaccine will offered at the end of the trial, depending on which vaccine the child initially received.

^g^Both the intervention and control arms will be asked to return for unblinding (at Day 730 only) and documentation of vaccination.

The proportion of participants developing adverse events within 7 days of vaccination and serious adverse events within 6 months of vaccination will be identified through both active follow-up contacts (telephone and in person) and self-reporting by participants.

An immunogenicity sub-study will investigate the immune response to the vaccine by drawing blood 4 times throughout the study: on Day 0, on Day 28, at 18 months, and at 2 years post-vaccination. These blood samples will also be used to investigate the duration and persistence of IgG Vi-antibodies, using enzyme-linked immunosorbent assay (ELISA) vacyme, over time.

### Outcomes

The primary outcome is the incidence of blood culture–confirmed typhoid fever.

Other secondary and exploratory outcomes include: inpatient/outpatient admission rates for fever; rates of hospitalization for febrile illnesses; paratyphoid infection rates; lengths of hospital stays due to febrile illnesses; antibiotic use; durations of febrile illnesses; rates of all-cause hospitalizations; children’s growth in height and weight (measured at the beginning and end of the study for children under 5 years at the time of enrollment); rates of absenteeism from school/work as the result of illnesses; rates of all-cause mortality; rates of acute abdominal presentations; and surgical interventions. These outcomes will be measured via passive surveillance, participant follow-up interviews, and hospital record reviews.

Blood samples, collected from suspected typhoid cases and from the immunogenicity sub-study, will be analyzed to investigate individual host’s genetic susceptibility to typhoid, genetic control of immunity to the vaccine, and susceptibility to infectious diseases like typhoid, in those with and without Vi-TCV. Blood samples will have DNA extracted, and the DNA will be whole-genome genotyped using Illumina genotyping arrays. Genome-wide data will be used for a quantitative trait locus analysis, to identify those genes that contribute to the Vi antibody.

### Sample Size

The study is powered for the primary endpoint. Sample size calculations are based on the following assumptions (Table 2):

**Table 2.** Sample Sizes Under Varying Assumptions in Nepal (80% Power, 5% Alpha)

Overall Incidence in the Entire Population Without Vaccination per 100 000	Direct Effect of Vaccine	Total Number to Enroll
100 cases py	80%	12 475
85 cases py	80%	14 677
100 cases py	75%	14 785
85 cases py	75%	17 395

Abbreviation: py, person-years.

1. An overall incidence of typhoid fever, in the absence of vaccination, of 85 cases per 100 000 people in the population per year, with 238 cases per 100 000 people per year in children under 16 years.
2. Age-specific incidence rates were determined from the age distribution of typhoid cases, which is specific to Kathmandu, from published estimates and from site-specific surveillance data.
3. A direct effect of vaccination of 75% and an indirect effect of 25%, based on mathematical modelling.
4. A 25% loss to follow-up per year, due to participants moving out of the area.

These assumptions are conservative; however, to allow for further variation in the assumptions, the total sample size has been increased to 20 000 children (10 000 in each vaccination arm).

Over the 2-year follow-up of the trial, if the assumptions are correct, approximately 36 cases of typhoid in the control arm and 9 cases in the Vi-TCV arm are expected to be seen.

### Data Analysis Plan

The incidence of typhoid will be estimated as the number of cases, divided by the total number of person-years of follow-up. The incidences will be presented with 95% CIs for each group and overall. An incidence rate ratio (IRR) will be computed as the ratio of the incidence in the Vi-TCV arm, compared to the MenA control arm. Vaccine efficacy will be calculated as (1–IRR) × 100%.

The cumulative incidence of typhoid will be summarized using the Kaplan-Meier method. Participants will be censored in the analysis at the time of their last-known residence in or near the surveillance area, at the last known contact time, or at the 2-year final visit. Statistical significance will be determined as a *P* value from a log-rank test of less than .05.

Subgroup analyses will include:

1. Age: <5 years and ≥5 years
2. Age: <2 years and ≥2 years
3. Sex: male and female

## OTHER ETHICS CONSIDERATIONS

All efforts will be made to conduct the research in a way that is sensitive to the Nepali culture and social values. Nepali trial staff will take consent, and the participant study-related materials (information sheet, consent forms, etc) will be printed in Nepali.

The MenA vaccine was selected as the control vaccine to ensure that the control group is receiving a beneficial intervention. The control vaccine will provide protection against group A meningitis, which is currently the most common capsular group in Nepal and can cause severe disease.

Both the intervention and control vaccines have been tested for safety in previous trials, are licenced for use in Nepal, and have received WHO prequalification. At the end of the trial, after unblinding, all participants will be offered the vaccine to which they were not randomized, allowing all participants to be vaccinated against both typhoid and meningococcal A.

For assays and analyses that are not available in Nepal, samples and data collected will be shared with other researchers in institutions outside of the country. Only anonymized samples and data will be sent outside of the research site. At the end of the study, all remaining samples in Nepal will be kept for storage in the Patan Hospital Microbiology Laboratory, as required by the Nepal Health Research Council. All samples will be kept for a minimum of 10 years after the end of the trial. These samples may facilitate important future research without needing new samples from Nepali children, should better tests become available.

Potential participants or their parents/legal guardians will be notified that they may refuse to have the relevant biological samples stored, without this influencing their study participation or the clinical care of their child. They will be informed that they may request that their samples be destroyed at any time, for any reason.

For the duration of the trial, the trial will cover the costs of standard care treatment for those participants presenting with fever ≥2 days, including the cost of tests, prescribed medications, and in-patient hospital care, as medically necessary. Incentives will be provided to children for all blood draws, in the form of school supplies.

The trial staff will ensure the maintenance of participants’ anonymity. Participants will be identified only by a participant ID number on all trial documents and electronic databases. All documents will be stored securely and will only be accessible by trial staff and authorized personnel. The trial will comply with the UK Data Protection Act, which requires data to be anonymized as soon as it is practical to do so, and with local regulations.

An independent Data and Safety Monitoring Board (DSMB) will be convened to act in an advisory capacity to the University of Oxford to monitor participant safety and data quality and to evaluate the progress of the trial. The DSMB will make recommendations to the University of Oxford about the continuation, modification, or termination of the trial, based on safety data shared with the members of the DSMB.

The protocol and all related trial materials have been approved by both the ethics committees and institutional review boards at the University of Oxford Tropical Research Ethics Committee (Oxford, UK) and the Nepal Health and Research Council (Kathmandu, Nepal).

## DISSEMINATION

The results of the study will be disseminated through a series of article publications, such that the findings will be available to the broader research communities. At the end of the trial, the results will also be shared with the participants, local leaders, and community members.

## CONCLUSION

This trial is the first large-scale, community-based, individually randomized, controlled trial of Vi-TCV in children in an endemic setting. The results of this trial will provide critical information about the impact of Vi-TCV in reducing typhoid infections in children, who bear a significant burden of the morbidity and mortality associated with this disease. These results will also support decision-making in many countries that are considering the introduction of Vi-TCV into routine childhood vaccination programs.

This protocol provides a clear outline of the planning and implementation necessary to conduct a high-impact, randomized, controlled trial in a low-resource setting. Researchers aiming to generate evidence of vaccine efficacy may find this protocol useful in understanding how trials of this nature can be designed.

## Notes

***Author contributions.*** K. T.-N. and M. S. developed the research protocol. R. C.-J. supported the clinical delivery plan and protocol finalization. M. V. wrote the analysis plan and conducted the statistical evaluation for the sample size. N. S. supported the protocol finalization. B. B., S. S., K. M. N., and A. J. P. provided senior research oversight and input into the research plan and protocol development.

***Acknowledgments.*** The authors thank the Bill & Melinda Gates Foundation for funding the Typhoid Vaccine Acceleration Consortium, including this trial; the Wellcome Trust and the Bill & Melinda Gates Foundation for funding of The Strategic Typhoid Alliance across Africa and Asia, which supported the surveillance that underpins this trial; and the National Institute for Health Research Oxford Biomedical Research Centre.

***Disclaimer.*** The views expressed in this manuscript are those of the authors and do not necessarily reflect the views of the Joint Committee on Vaccination and Immunisation, the National Health Service, the National Institute for Health Research, the UK Department of Health and Social Care, or the World Health Organization. The findings and conclusions contained within are those of the authors and do not necessarily reflect the positions or policies of the Bill & Melinda Gates Foundation.

***Financial support.*** This publication is based on research funded in part by a grant from the Bill & Melinda Gates Foundation (OPP1151153).

***Supplement sponsorship.*** This supplement is sponsored by the Center for Vaccine Development and Global Health (CVD) at the University of Maryland School of Medicine.

***Potential conflicts of interest.*** A. J. P. is a National Institute for Health Research Senior Investigator; chairs the UK Department of Health and Social Care’s Joint Committee on Vaccination and Immunisation; is a member of the World Health Organization’s Strategic Advisory Group of Experts; and reports grants from Pfizer and Okairos, outside the submitted work. K. M. N. is a member of the World Health Organization Strategic Advisory Group of Experts on Immunization. All other authors report no potential conflicts. All authors have submitted the ICMJE Form for Disclosure of Potential Conflicts of Interest. Conflicts that the editors consider relevant to the content of the manuscript have been disclosed.
